# The impact of disease-related immobilization on thigh muscle mass and strength in older hospitalized patients

**DOI:** 10.1186/s12877-020-01873-5

**Published:** 2020-11-25

**Authors:** Nikola Rommersbach, Rainer Wirth, Gero Lueg, Christiane Klimek, Mirja Schnatmann, Dieter Liermann, Gregor Janssen, Manfred James Müller, Maryam Pourhassan

**Affiliations:** 1 Department of Geriatric Medicine, Marien Hospital Herne, Ruhr-Universität Bochum, Hölkeskampring 40, 44625 Herne, Germany; 2 Department of Radiology, Marien Hospital Herne, Ruhr-Universität Bochum, Herne, Germany; 3 Institute of Human Nutrition and Food Science, Christian-Albrechts University, Kiel, Germany

**Keywords:** Muscle mass, Muscle strength, Adipose tissue, Intermuscular fat, Immobilization

## Abstract

**Background:**

We assessed the quantitative changes in muscle mass and strength during 2 weeks of hospitalization in immobile and mobile acutely ill hospitalized older adults.

**Methods:**

Forty-one patients (82.4 ± 6.6 years, 73.0% females) participated in this prospective longitudinal observational study. Mobility status was defined according to walking ability as described in the Barthel-Index. Functional status, including handgrip strength and isometric knee-extension strength, and mid-thigh magnetic resonance imaging (MRI) measurements of cross-sectional area (CSA) were conducted on admission and at discharge.

**Results:**

Twenty-two participants (54%) were immobile and 19 (46%) mobile. In all, 54.0 and 12.0% were at risk of malnutrition and malnourished, respectively. The median time between baseline and follow-up for MRI scans were 13 days in mobile and immobile participants (*P* = 0.072). Mid-thigh muscle and subcutaneous fat CSA significantly decreased by 3.9cm^2^ (5.0%, *P* = 0.002) and 5.3cm^2^ (5.7%, *P* = 0.036) during hospitalization whereas intermuscular fat remained unchanged in immobile subjects. No significant changes were observed in mobile patients. In a regression analysis, mobility was the major independent risk factor for changes in mid-thigh muscle CSA as a percentage of initial muscle area (*P* = 0.022) whereas other variables such as age (*P* = 0.584), BMI (*P* = 0.879), nutritional status (*P* = 0.835) and inflammation (*P* = 0.291) were not associated with muscle mass changes. There was a significant decrease in isometric knee extension strength (*P* = 0.002) and no change in handgrip strength (*P* = 0.167) in immobile patients whereas both parameters increased significantly over time in mobile patients (*P* = 0.048 and *P* = 0.012, respectively).

**Conclusions:**

Two weeks of disease-related immobilization result in a significant loss of thigh muscle mass and muscle strength in older patients with impaired mobility. Concomitantly, there was a significant reduction of subcutaneous adipose tissue in immobile older hospitalized patients whereas no changes were observed in intermuscular fat among these patients. These data highlight the importance of mobility support in maintaining muscle mass and function in older hospitalized patients.

## Background

Alterations in body composition with advancing age have important implications for functional status, health and survival. The progressive loss of muscle mass, muscle strength and physical performance is a part of the aging process. It affects individuals from almost the age 50 years with an annual rate of decline in muscle mass and muscle strength by 1 and 3%, respectively [1–3]. However, the magnitude and severity of decline in muscle mass and muscle strength may depend on health compromising behaviors such as physical inactivity and nutritional difficulties [4, 5] and may further deteriorate by disease and disease-related immobilization [6]. The term sarcopenia was employed to describe patients with compromised muscle mass and function and define clinically relevant threshold values for muscle mass, strength and function [6].

The presence of sarcopenia is associated with adverse health outcomes such as physical disability, falls, frailty, hospital admissions and mortality [3, 7]. In our recent study [8] among 198 older hospitalized patients (mean age 82.8 ± 5.9 years), after adjustment for potential confounders such as age and gender, sarcopenia was associated with increased 1-year mortality among patients with limited mobility prior to admission (*n* = 138, hazard ratio, HR: 2.52, 95% CI: 1.17–5.44) and at time of discharge (*n* = 162, HR: 1.93, 95% CI: 0.67–3.22). In another prospective cohort study in persons aged over 80 years living in the community, Landi et al. [9] reported that patients with sarcopenia had a higher risk of death during the 10-year follow-up than those without sarcopenia (HR = 2.15; 95% CI: 1.02–4.54). In addition to physiological age-related causes of sarcopenia, reduced mobility, low food intake and inflammation also play an important role [10, 11]. Indeed, the interplay between acute diseases and factors such as immobility and malnutrition, all prevalent among older adults, may superimpose and accelerate the process of muscle loss in this population [12, 13].

A variety of conditions, such as falls and fractures, surgical interventions, acute diseases and hospitalization lead to immobilization, muscle disuse and functional decline [11, 14], which may further aggravate the loss of muscle mass and muscle strength [11, 15, 16]. Previous bed rest studies reported that healthy older adults lost approximately 4–6% of total lean leg mass following seven to ten days of immobility [17, 18]. However, these studies investigated changes in lean mass among healthy older adults using experimental models of immobilization. Still, very little is known about the consequences of disease-related immobilization on body composition and muscle among immobile frail older hospitalized patients. Recently, Kouw et al. [19] indicated that 1 week of hospitalization following elective hip surgery resulted in substantial loss in thigh muscle mass of the unaffected leg by 4.2%, as measured by computed tomography, in older hospitalized patients. However, it is of great importance to determine the effect of immobility during hospitalization not only on muscle mass, but also on subcutaneous fat and its role in metabolic changes [20] and on intermuscular fat as a potential contributor to decreasing muscle strength and muscle quality in older individuals [21–23]. Beyond muscle mass, adipose tissue (e.g. intermuscular fat) has also significant role in mobility limitation in older adults and may influence muscle health and quality [24]. Results of previous studies among older individuals demonstrated a close association between adipose tissue and muscle mass function and mobility [25, 26]. Therefore, measurement of fat mass should be also considered in preserving muscle strength and muscle quality in old age. In addition, it is essential to investigate the impact of muscle mass loss on changes in muscle strength as the most relevant parameter for functional limitation. To the best of our knowledge, no studies have specifically examined such associations among acutely ill immobile older hospitalized patients up to now.

Magnetic resonance imaging (MRI) is the gold standard for assessment of body composition, although its application is limited due to time-consuming assessment of whole-body tissue volumes and high costs [27–29]. Therefore, several studies have suggested to estimate muscle volumes from a single-slice section at mid-thigh [29–31], since lower limb power has been considered as a critical factor for mobility in older adults [32]. With the assumption that thigh muscles correspond to spheroids, any change in cross-sectional area is proportional to the respective change in volume. The aim of this study was to examine the impact of 2-weeks disease-related immobilization on muscle mass and fat mass among acutely ill older hospitalized patients. Accordingly, we assessed the quantitative changes in muscle, subcutaneous and intermuscular fat cross-sectional area using a single-slice MRI at mid-thigh and compared the data with a healthier and mobile group of older patients during hospitalization as a reference group.

## Methods

This prospective observational study was performed at the geriatric department of our university hospital. A detailed description of the methods has been reported elsewhere [33]. Mobility status was evaluated according to walking ability as described by the respective item of the Barthel-Index (BI) [34] and patients were grouped into two categories as mobile group (walking ability score of 15 or 10) and immobile group (walking ability score of 5 or 0).

It is worth noting that mobile and immobile patients were selected from the geriatric day clinic and the geriatric hospital department, respectively. Geriatric day clinic is a facility in between ambulatory and in-patient treatment, i.e. patients stay for 7–8 h every day for several days and get their diagnostic procedures and treatment. The remaining time they stay at home, which implies they are more or less mobile and able to take care for themselves. Indeed, patients from the geriatric day clinic had better functional and nutritional status compared to those from the geriatric hospital department. In general, the patients who are admitted to the geriatric hospital department are really ill and frail and sometime stay even more than 16 days in hospital based on their clinical situation.

The inclusion criteria for participation of both groups were patients of 65 years or older who were expected to be hospitalized for at least 14 days, ability to cooperate and written informed consent. Exclusion criteria were immobility longer than 3 weeks before recruitment, leg amputation, pacemaker implants and severe disturbance of fluid status. Barthel-Index, measurement of muscle strength, body weight and mid-thigh MRI measurements were conducted within 24 h after hospital admission (baseline) and before discharge (follow-up). In addition, geriatric assessment was performed and C-reactive Protein (CRP) was analyzed according to standard clinical procedures at hospital admission. The study protocol had been approved by the ethical committee of Ruhr-University Bochum (17–6048, approved on 08.08.17).

Self-caring ability and functional status were determined using Barthel-Index (BI) [34], the FRAIL scale [35] and the SARC-F questionnaire [36]. The number and severity of medical comorbidities were classified with the Charlson Comorbidity Index (CCI) [37].

Nutritional status was evaluated using the Mini Nutritional Assessment Short Form (MNA-SF) [38] and food intake was determined using the semi-quantitative plate diagram method [39].

Body weight was assessed in light clothing with an accuracy of 0.1 kg and height was measured to the nearest 0.5 cm with a stadiometer during hospitalization. The degree of unintentional weight loss prior to admission was obtained either by interviewing the patients, if competent, or asking their proxy, where necessary.

Irrespective of mobility status, similar nutrition was provided to all patients except oral nutritional supplements which were only provided for malnourished patients. Physical therapy for at least 30 min twice a day was offered to all patients as a routine rehabilitation program. However, immobile patients who were more or less bedridden participated less. Furthermore, all patients had an individualized training program according to the deficiencies in activities of daily living.

**Fig. 1:**
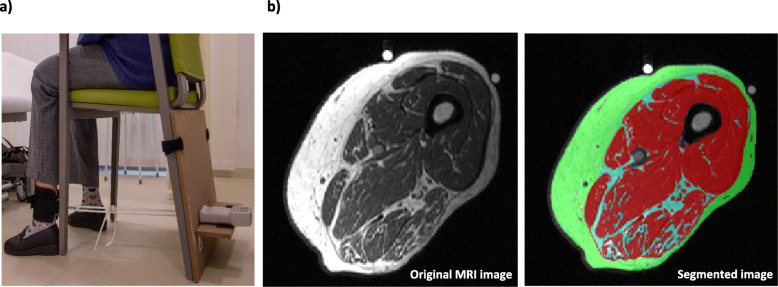
**a** Maximal isometric knee extension strength measurement and **b** A sample of single-slice mid-thigh MRI image of a 68-year-old female, mobile patient before and after segmentation. Structures in red: muscle, green: subcutaneous fat, blue: intermuscular fat

The protocol described by Gandevia [40] and a Jamar dynamometer were used to assess isometric knee extension strength and hand grip strength (HGS) respectively. Knee strength was measured while sitting on a chair with a strap around the distal leg (Fig. 1a). The best of three attempts was recorded.

MRI scans were performed with a Siemens Magnetom Sonata, 1,5 Tesla (Siemens Medical Solutions, Erlangen, Germany) at a reproducible position of mid-thigh to quantify muscle, subcutaneous and intermuscular fat cross-sectional area (CSA). The details of the procedure including the segmentation are described elsewhere [33].

### Statistical analysis

The statistical analysis was performed using SPSS statistical software (SPSS Statistics for Windows, IBM Corp, Version 24.0, Armonk, NY, USA). Continuous variables are expressed by their means and standard deviations (SDs) or median values with interquartile ranges (IQR), as appropriate. Categorical variables are expressed as n (%). Differences between immobile and mobile groups were analyzed by using paired samples *t* test for normally distributed values. The magnitude of changes at follow-up between groups were analyzed by using an unpaired *t* test in normally distributed variables and the Mann-Whitney U test for continuous variables with non-normal distribution. Chi square test was used to compare categorical variables. A stepwise multiple regression analysis was used to examine the impact of risk factors such as mobility status, age, BMI, malnutrition and inflammation (as independent variables) on changes in mid-thigh muscle CSA as a percentage of initial muscle area as (dependent variable). *P* < 0.05 was determined as the limit of significance.

## Results

### Subject characteristics

Baseline characteristics of study participants stratified by mobility status are summarized in Table 1. Of 41 patients with a mean age of 82.4 ± 6.6 years (73.0% females), 22 (54%) were immobile (64.0% females) and 19 (46%) were mobile (84.0% females). In total study population, 34.0 and 54.0% had normal nutritional status or a risk of malnutrition, respectively whereas 12.0% were malnourished according to MNA-SF. Major reasons for hospitalization were 59.0% musculoskeletal diseases including fractures (e.g. vertebral, femoral and pelvic fractures), 15.0% gait disorders and 26.0% various general diseases such as heart failure, stroke and infectious diseases. Twelve of the immobile patients underwent surgery prior to the admission to the geriatric ward. In addition, the reasons for hospitalization in the immobile group were similar to the entire population with the corresponding values of 57.0, 14.0 and 29.0%, respectively.

**Table 1:** Characteristic of study population at baseline (T0) stratified by mobility status

	All (*n* = 41)	Immobile group (*n* = 22; 54%)	Mobile group (*n* = 19; 46%)
Female	30 (73.0)	14 (64.0)	16 (84.0)
Male	11 (27.0)	8 (36.0)	3 (16.0)
Age (y)	82.4 ± 6.6	82.5 ± 6.5	82.2 ± 6.8
Height (m)	1.61 ± 0.1	1.64 ± 0.1	1.58 ± 0.1*
BMI (kg/m^2^)	28.4 ± 6.4	26.3 ± 4.8	30.7 ± 7.3*
Weight loss in 6 months (kg)	2.2 ± 3.1	2.6 ± 3.0	1.7 ± 3.3
CRP (mg/dl)	2.5 ± 5.5	4.1 ± 7.1	0.6 ± 1.0*
MNA-SF score, Median (IQR)	10 (8–12)	9 (7–10)	12 (11–13)***
Normal nutritional status (n; %)	14 (34.0)	1 (4.0)	13 (68.0)
At risk of malnutrition (n; %)	22 (54.0)	16 (73.0)	6 (32.0)
Malnourished (n; %)	5 (12.0)	5 (23.0)	0 (0.0)
Barthel-Index on admission, Median (IQR)	55 (40–67)	40 (35–51)	70 (60–80)***
Frail Simple scale score, Median (IQR)	3 (2–3)	3 (2–3)	3 (2–3)
*SARC-F* scores, Median (IQR)	6 (4–7)	7 (5–8)	5 (2–6)**
CCI score, Median (IQR)	2 (1–3)	2 (1–3)	2 (1–2)
Handgrip strength (kg)	19.8 ± 8.3	20.1 ± 8.3	19.4 ± 8.5
Isometric knee extension strength (kg)	16.6 ± 6.7	16.4 ± 6.9	17.0 ± 6.8
Mid-thigh MRI cross sectional area (cm^2^)			
Muscle area	81.2 ± 18.2	78.7 ± 17.3	84.0 ± 19.3
Subcutaneous fat area	89.4 ± 53.2	80.0 ± 44.4	100.3 ± 61.3
Intermuscular fat area	18.1 ± 9.5	17.1 ± 9.0	19.2 ± 10.3

*CRP* C-reactive protein, *MNA-SF* Mini Nutritional Assessment Short Form (normal nutritional status 12–14 points, at risk of malnutrition 8–11 points and malnourished 0–7 points); Frail Simple scale (not frail with score 0, pre-frail with scores of 1–2 and frail with 3–5); SARC-F scores (high risk of sarcopenia with score ≥ 4); *CCI* Charlson Comorbidity Index, *MRI* Magnetic Resonance Imaging. Values are given as mean ± SD, number (%) or median (IQR, interquartile range). There were no significant differences in gender distribution between the mobile and immobile groups (*P* = 0.138). *P < 0.05, **P < 0.01, ***P < 0.001, Difference between immobile and mobile patients (unpaired t test)

The patients in the immobile group were taller (*P* = 0.037), had lower BMI (*P* = 0.032) and Barthel-Index (*P* < 0.001) and showed a higher prevalence of poor nutritional status (*P* < 0.001) at baseline than the mobile group. In addition, frailty was present in immobile and mobile patients (*P* = 0.087) and both groups had probable sarcopenia according to SARC-F with higher median value in immobile patients (*P* = 0.004). At baseline, the mean CRP level was 2.5 ± 5.5 mg/dl in total population indicating moderate inflammation. The mean CRP level was significantly higher in immobile (4.1 ± 7.1 mg/dl) than in mobile patients (0.6 ± 1.0 mg/dl; *P* = 0.030). Furthermore, immobile and mobile groups did not significantly differ at baseline for handgrip strength, knee extension strength and mid-thigh CSA (Table 1).

No statistically significant differences either in average length of stay (*P* = 0.208) or in time between baseline and follow-up MRI scans (*P* = 0.072) between mobile and immobile patients were observed. The median time from baseline to follow-up for MRI scan was 13 days in both mobile (IQR: 12–15) and immobile groups (IQR: 10–14).

### Comparison of CSA of MRI

**Fig. 2:**
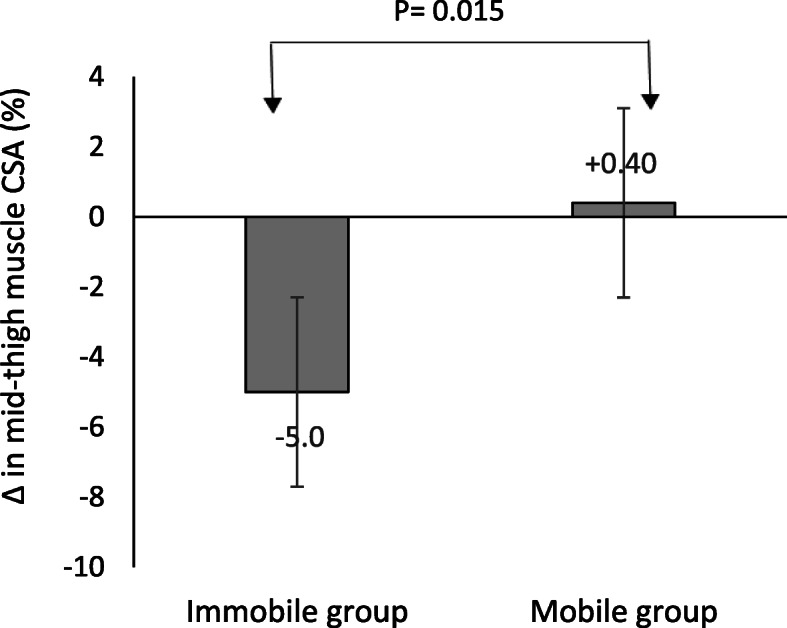
Comparison of changes in mid-thigh muscle cross-sectional area (CSA) as a percentage of initial muscle area between immobile (*n* = 22) and mobile patients (*n* = 19; unpaired t test) after 13 days of hospitalization

Detailed results for mid-thigh MRI-CSA stratified by mobility status at baseline and follow-up and respective changes during hospitalization are shown in Table 2 and Fig. 2. At baseline, no significant differences in mean mid-thigh CSA of muscle, subcutaneous and intermuscular fat between the immobile and mobile group were found. By contrast, mean mid-thigh muscle CSA significantly decreased by 3.9 cm^2^ (5.0%) during hospital stay in immobile patients (*P* = 0.002) and remained unchanged in mobile patients (*P* = 0.717). In addition, changes in mid-thigh muscle CSA per day was significantly higher in immobile patients compared to the mobile group (− 0.3 cm^2^ vs. + 0.1 cm^2^; *P* = 0.013, respectively; Table 2). In a stepwise regression analysis, mobility was the major independent risk factor for changes in mid-thigh muscle CSA as a percentage of initial muscle area (*P* = 0.022) whereas other variables such as age (*P* = 0.584), BMI (*P* = 0.879), MNA-SF (*P* = 0.835) and CRP level (*P* = 0.291) did not show any impact on muscle mass changes.

**Table 2:** Comparison of mean mid-thigh MRI cross sectional area (cm2), body weight and Barthel-Index of study population stratified by mobility status at baseline (T0) and follow-up (T1)

	Immobile group (*n* = 22)				Mobile group (*n* = 19)			
Mid-thigh CSA (cm^2^)	T0	T1	ΔT1–T0	Δ/day	T0	T1	ΔT1–T0	Δ/day
Muscle area	78.7 ± 17.3	74.8 ± 17.9	−3.9 ± 5.0**†	−0.3 ± 0.4††	84.0 ± 19.3	84.5 ± 20.6	0.5 ± 5.6	0.1 ± 0.5
Subcutaneous fat area	80.0 ± 44.4	74.7 ± 40.0	−5.3 ± 11.1*	−0.5 ± 1.0	100.3 ± 61.3	97.9 ± 56.9	−2.4 ± 18.2	−0.1 ± 1.3
Intermuscular fat area	17.1 ± 9.0	16.0 ± 8.9	−1.1 ± 2.9	−0.1 ± 0.2	19.2 ± 10.3	19.4 ± 12.1	0.2 ± 3.5	0.0 ± 0.3
Body weight (kg)	70.9 ± 14.4	70.1 ± 14.9	−0.8 ± 2.5	−0.05 ± 0.2	77.2 ± 19.0	75.8 ± 19.0	−1.5 ± 7.1	−0.1 ± 0.5
Barthel-Index	42.3 ± 11.4	61.8 ± 16.8	19.5 ± 13.0***†	1.4 ± 1.0	68.7 ± 11.9	81.3 ± 8.8	12.6 ± 8.7***	0.9 ± 0.6

All values are means ± SDs. No significant group difference in time between baseline and follow-up of MRI scan was observed (*P* = 0.072). The median time of follow-up for MRI scan was 13 days in both mobile (IQR: 12–15) and immobile groups (IQR: 10–14)

*CSA* cross sectional area, *MRI* Magnetic Resonance Imaging, *ΔT1–T0* difference between baseline and follow-up, *Δ/day* change per day

*P < 0.05, **P < 0.01, ***P < 0.001 Difference between T0 and T1 within group (paired t test)

†*P* < 0.05 Difference in ΔT1–T0 between groups (unpaired t test)

††*P* < 0.05 Difference in Δ/day between groups (unpaired t test)

Moreover, evaluation of mid-thigh CSA of subcutaneous fat identified a statistically significant decline of 5.3 cm^2^ (5.7%) in immobile patients only (*P* = 0.036). Concomitantly, absolute mid-thigh CSA of intermuscular fat and body weight remained unchanged over time in both groups (Table 2). Furthermore, Barthel-Index substantially improved during hospitalization in immobile and mobile groups (both *P* < 0.001) with significantly more improvement in immobile patients (*P* = 0.05, Table 2).

There was a significant decline of 12% in isometric knee extension strength (T0: 16.6 kg, T1: 14.5 kg; *P* = 0.002) and nearly no change in handgrip strength (T0: 20.1 kg, T1: 19.1 kg; *P* = 0.167) in immobile patients during hospitalization whereas knee extension (T0: 16.9 kg, T1: 18.1 kg; *P* = 0.048) and handgrip (T0: 19.4 kg, T1: 20.8 kg; *P* = 0.012) strength increased significantly over time in mobile patients. Further, no significant differences in the amount of food intake were observed between mobile and immobile groups (*P* = 0.196).

## Discussion

The major finding of the present study is that almost 2 weeks of disease-related immobility result in significant thigh muscle mass loss of 5.0% in a group of immobile older patients admitted to an acute care geriatric unit, while such an effect was not seen among mobile older patients. Notably, this substantial reduction occurred despite providing the medical support and training therapy offered in geriatric units. It can only be speculated how much muscle mass and strength would have been lost without this support. The detrimental impact of bed rest on muscle mass and strength has been already reported in previous experimental models of immobilization in healthy older adults. Studies using muscle mass disuse model have shown approximately 2–6% reduction of leg muscle mass following 5 to 14 days among this population [16, 18, 41].

However, only few published studies have investigated the actual muscle mass loss in a clinical setting. Namely, the effect of immobilization on muscle mass remains to be elucidated in acutely ill older hospitalized patients who are likely to experience a more pronounced loss of muscle mass due to their condition. Hospitalization is commonly accompanied by enforced bedrest or poor mobility induced by pain, surgical trauma, infections and mental stress, leading to changes in food intake and skeletal muscle catabolism [19, 42, 43]. Consequently, we hypothesized that even a short period of hospital stay may result in a significant loss of muscle mass and strength in patients with severe mobility limitation. In a recent observation study in older patients undergoing elective hip replacement, Kouw et al. [19] reported a significant loss of thigh muscle CSA by 4.2% ± 1.1% (0.6% per day) using CT scan during one-week hospitalization. Our findings were similar, although our patients were older and experienced 5.0% reduction in MRI-derived mid-thigh muscle CSA during the course of a 13-day hospitalization period (0.4% per day). Notably, disease-related immobilization in our cohort started up to 3 weeks before admission to our department and thus before the first MRI scan. In line with our findings and the aforementioned study, we assume that within the first days of immobilization, the rate of muscle mass loss may be even higher than in our study and may decrease over time, due to metabolic adaptations.

In another prospective study of 63 critically ill patients (mean age 54.7 years), Puthucheary et al. [44] has indicated the significant decline in the ultrasound-derived rectus femoris CSA by 17.7% at day 10 of hospitalization. However, the changes in muscle mass were greater compared to our findings. Although, direct comparison is difficult since that study used a different method for assessing muscle mass and patients were younger and suffered from multi-organ failure. Nevertheless, it has to be noted that in that study reduction of muscle mass was more severe among those with multi-organ failure compared with single organ failure. Indeed, despite the fact that immobilization is a factor in development of muscle atrophy, patients with multi-organ failure may develop more muscle mass loss as a result of greater metabolic changes [45, 46] and other deleterious factors associated with severe disease, rather than immobility alone [44]. In the present study, despite the similar mid-thigh muscle CSA in both mobile and immobile older adults at baseline, the MRI scans, which provide a very sensitive and accurate measurement, clearly indicated the substantial decline in muscle mass only in immobile older patients during hospitalization. Moreover, the majority of our immobile patients were frail, probably sarcopenic and were at risk of malnutrition or malnourished at the time of admission. Therefore, the combination of these factors may have affected the extent of muscle mass loss in our study [19, 42, 43].

In addition, prior researches on the morphologic changes associated with immobility in older adults have commonly concentrated on muscle mass whereas changes in subcutaneous or intermuscular fat have not received a great deal of attention. However, this knowledge is important since alterations in adipose tissue are linked with dysfunction and metabolic changes in skeletal muscle [47, 48]. Indeed, interaction between adipose tissue and muscle mass is influenced by mobility and aging. Mobility limitation caused by aging leads to decline in muscle mass and function and alteration in body fat composition [24]. With advancing age, intermuscular adipose tissue increases [24] and subcutaneous tissue decreases [49]. These significant changes in fat composition may have a negative impact on health outcome in old age. Fatty infiltration of the skeletal muscle is a metabolically active component of muscle and affects muscle strength and muscle quality [50]. It secretes inflammatory cytokines which negatively impact muscle cell proliferation and differentiation [50]. The findings of the present study demonstrated a significant decline in mid-thigh subcutaneous fat area in immobile patients without changes in intermuscular fat. It has been previously shown that immobilization leads to increased intermuscular fat [51, 52]. For instance, intermuscular adipose tissue of thigh increased in healthy young patients during 4 weeks of immobilization [51] and in patients with spinal cord injury [52]. However, this could not be shown in the period of 13 days of immobilization in our study. This discrepancy could be a result of differences in the study populations and in length of the follow-up period. Since the current sample included ill older adults with several risk factors such as malnutrition, frailty and severe disease, immobilization may specifically and differentially affect both adipose tissues in our population. From a metabolic point of view, our findings indicate that subcutaneous and intermuscular fat may have structural and functional differences in response to immobilization and are subject to distinct dysfunctional changes caused by disease, aging and lifestyle. Indeed, subcutaneous adipose tissue is metabolized during periods of immobilization and decreased nutritional intake and seems to be metabolically more active compared to intermuscular fat. Moreover, previous cross-sectional studies have demonstrated that a greater fat infiltration of the muscle is an independent risk factor for mobility limitations and is a potential contributor to decreasing muscle strength and muscle quality in older individuals [1, 23].

Our results indicate a significant decrease in isometric knee extension strength and nearly no change in handgrip strength during hospitalization of acutely immobile patients. Accordingly, it could be that even short periods of immobilization do not only influence muscle mass but may have also negative effects on muscle strength and physical functioning of lower extremity of older adults and are likely to contribute to impaired recovery, increased readmissions and a higher mortality rate after discharge [53]. Interestingly, the distinct loss of lower and upper extremity strength demonstrates that disease-related immobility has a more pronounced effect on leg muscle strength compared to hand grip strength. Therefore, muscle strength of the leg seems to be the most relevant parameter for functional decline and can reflect mobility limitation whereas upper muscle strength is more related to general body composition. Hence, measurements of leg strength should receive more priority compare to hand grip strength, especially when studying older persons.

Some limitations of the study need to be addressed. Mobility status was defined according to walking ability as described by the BI, which may be imprecise. Nevertheless, a previous study in patients with stroke [54] has demonstrated that measurement of mobility as measured by the BI is reliable and agreement was generally high for total BI and walking ability. In addition, there was a shorter follow-up period of MRI scans for some patients during hospitalization, mostly due to organizational issues. However, this did not differ between both groups. Finally, due to the relatively small number of immobile patients (*n* = 22), we were unable to reliably examine the individual contribution of risk factors such as disease severity, malnutrition, surgical trauma, inflammation and medication to the actual loss of muscle mass. Thus, this highlights the necessity to establish further studies to address the impact of individual risk factors on the extent of muscle mass loss in the clinical setting.

## Conclusion

We conclude that almost 2 weeks of disease-related immobilization result in a significant loss of thigh muscle mass and muscle strength in older patients with impaired mobility. Concomitantly, there was a significant reduction of subcutaneous adipose tissue in immobile older hospitalized patients whereas no changes were observed in intermuscular fat among these patients. These data should highlight the importance of mobility support in maintaining muscle mass and function in older hospitalized patients.

## Data Availability

The datasets used and analyzed during the current study available from the corresponding author on reasonable request.

## Abbreviations

CSA: Cross-sectional area
MRI: Magnetic Resonance Imaging
BI: Barthel-Index
CRP: C-reactive Protein
MNA-SF: Mini Nutritional Assessment Short Form
CCI: Charlson Comorbidity Index
